# Mu Suppression Is Sensitive to Observational Practice but Results in Different Patterns of Activation in Comparison with Physical Practice

**DOI:** 10.1155/2018/8309483

**Published:** 2018-05-27

**Authors:** Najah Alhajri, Nicola J. Hodges, Jill G. Zwicker, Naznin Virji-Babul

**Affiliations:** ^1^Graduate Program in Rehabilitation Sciences, University of British Columbia, Vancouver, BC, Canada; ^2^School of Kinesiology, Faculty of Education, University of British Columbia, Vancouver, BC, Canada; ^3^Department of Occupational Science & Occupational Therapy, University of British Columbia, Vancouver, BC, Canada; ^4^BC Children's Hospital Research Institute, Vancouver, BC, Canada; ^5^Department of Physical Therapy, University of British Columbia, Vancouver, BC, Canada; ^6^Djavad Mowafaghian Centre for Brain Health, University of British Columbia, Vancouver, BC, Canada

## Abstract

Research has shown the effectiveness of observational practice for motor learning, but there continues to be debate about the mechanisms underlying effectiveness. Although cortical processes can be moderated during observation, after both physical and observational practice, how these processes change with respect to behavioural measures of learning has not been studied. Here we compared short-term physical and observational practice during the acquisition and retention of a novel motor task to evaluate how each type of practice modulates EEG mu rhythm (8–13 Hz). Thirty healthy individuals were randomly assigned to one of three groups: (1) physical practice (PP), (2) observational practice (OP), and (3) no practice (NP) control. There were four testing stages: baseline EEG, practice, postpractice observation, and delayed retention. There was significant bilateral suppression of mu rhythm during PP but only left lateralized mu suppression during OP. In the postpractice observation phase, mu suppression was bilateral and larger after PP compared to that after OP. NP control showed no evidence of suppression and was significantly different to both the OP and PP groups. When comparing the three groups in retention, the groups did not differ with respect to tracing times, but the PP group showed fewer errors, especially in comparison to the NP group. Therefore, although the neurophysiological measures index changes in the OP group, which are similar but moderated in comparison to PP, changes in these processes are not manifest in observational practice outcomes when assessed in a delayed retention test.

## 1. Introduction

A common instructional method in the teaching of motor skills has been to watch repeated demonstrations with the intention of later reproduction, so termed observational practice or observational learning (for recent reviews regarding its relative efficacy, see [1–6]). Many individuals, perhaps as a result of injury, neurological impairment, or fatigue, cannot engage in physical practice, at least all of the time, such that learning through observation serves as an alternative practice method for motor gains [7]. Despite the potential efficacy of this approach, at least in comparison to no-practice conditions, the mechanisms underpinning its efficacy are still debated, as well as its relative benefits in comparison to actual physical practice. In this paper, we evaluate the similarities and differences between observation and physical practice, in terms of both behavioural performance and learning outcomes as well as neurophysiological processes assessed through EEG and mu rhythm suppression.

We now know that watching others perform skills that are part of their existing motor repertoire engenders similar cortical neural processes to those apparent during actual action execution. There is a considerable body of research pointing towards a motor simulation circuit or mirror-neuron system in the human brain that responds in a similar way to observed and executed movements, supporting the idea of a shared neural representation between action observation and action execution (for reviews, see [8–11]). There are a wide range of brain areas that are activated during both observation and execution of actions, with the primary regions including the ventral and dorsal premotor cortex (PMC) [12–16], the intraparietal cortex [13, 17] and the superior and inferior parietal lobule [14, 15, 18]. For a recent review of these areas, see a meta-analysis by Caspers et al. [19].

One index of mirror neuron activity that has been extensively studied in humans is mu (8–13 Hz) suppression. At rest, neurons in the sensorimotor area fire synchronously, resulting in large-amplitude EEG and MEG oscillations in the mu frequency band. When participants perform an action, imagine movement, or observe movements, these neurons fire asynchronously, decreasing the power of the mu band [20–25]. This suppression is known as alpha-band or mu-rhythm suppression (also related to event-related desynchronization (ERD)). It has been hypothesized that the mu rhythms reflect downstream modulation of primary sensorimotor areas by mirror neuron activity, representing a critical information processing function translating perception into action [26]. When comparisons have been made across execution and observation conditions, suppression is typically stronger in execution conditions [23]. For very simple movements (e.g., repetitive finger pointing [20]), as well as more functional movements (e.g., reaching and grasping a coffee cup [24]), bilateral suppression, particularly in central electrode sites, has been noted. Importantly, Virji-Babul et al. [27] showed that although both conditions activate similar brain areas, there were distinct differences in the timing and pattern of the activation. During movement execution, the earliest activation was observed in the left premotor and somatosensory regions, followed closely by left primary motor and superior temporal gyrus (STG) at the time of movement onset. In contrast, during observation, there was a shift in the timing of activation with the earliest activity occurring in the right temporal region followed by activity in the left motor areas, suggesting that there are important differences underlying the neural processes of action execution and action observation.

While there is now a significant body of literature linking perception and action in well-learned actions, studies looking at processes underpinning observation practice of new actions are still rather scarce. With respect to behavioural work, generally it has been shown that observation and physical practice can lead to similar behavioural (though weaker in observation) motor outcomes [4, 28, 29] (for a meta-analysis see Ashford et al. [30]). However, notable differences have also been reported. For example, although people learn from watching others perform novel visuomotor adaptation tasks, where people learn to move to radially aligned targets that are rotated from their veridical spatial position, observers do not show after-effects that are nearly always seen in physical practice participants [5, 31]. The absence of after-effects after observational practice has led to conclusions that the two modes of learning implicate distinct brain networks, with an absence or reduction in motor-system (implicit) adaptation following only observation practice.

There is also evidence supporting the idea that observation and physical practice involve similar “motor-related” mechanisms and networks. For example, in studies of sequence learning, observation learning effectiveness was specific to the observed hand [29, 32], suggesting effector-specific modulation of the motor system during observation. In studies where observers watched a learner adapt to a dynamic perturbation controlled by a robot arm, at least some of the observation learning effects appeared to be mediated by the observer's motor system (e.g., [33, 34]). This was shown through dual-task interference effects associated with performing a motor task during the observation practice phase as well as interference from a postobservation practice period of repetitive TMS (transcranial magnetic stimulation) to the primary motor cortex (M1).

There have been two general hypotheses concerning the transfer of information during observation learning [35]. The first is that information is primarily cognitively mediated (also termed late mediation), such that the motor system does not play a role in learning until the later physical enactment stage. The second is a primarily motor-mediated learning (also known as early mediation), whereby observation is thought to automatically activate the motor representations of the observed action in the observer's brain. This motor resonance or simulation is thought to allow for action understanding, and hence learning, to occur [35, 36]. Behavioural studies provide support for both proposals, suggesting that information transfer during observational practice could be a result of either or both processes. There have been no studies to date where measurement of both the neurophysiological and behavioural processes of observation and physical practice of the same task has been determined concurrently during both types of practice.

In relation to observational practice, Nakano et al. [37] recorded EEG signals during the observation, preparation, and execution of five trials of a two-ball-rotation task. Across all three conditions, mu suppression in the fifth trial was significantly greater than that in the first trial. However, no comparisons were made between a pure observation practice-only group and a physical practice group (i.e., after the first trial, the second observation phase was a combination of both observation and execution) and no efforts were made to assess learning, as based on a retention or transfer test [38, 39].

Neurophysiological responses have been probed during action observation under conditions where motor/physical practice experiences have already been attained. For example, EEG was recorded in professional dancers and nondancers while they watched video clips of dance movements and everyday movements. Expert dancers exhibited significantly more mu suppression compared to nondancers, with no difference between the two groups during the observation of everyday movements [40] (see also [41, 42]). However, Babiloni et al. [43] reported that long-term experience was associated with less mu rhythm suppression in action-observation-related areas during the observation of familiar actions.

Despite evidence that physical experience modulates a subsequent observation phase with respect to EEG activity, to date, no researchers have studied how visual or physical/motor experiences with a novel motor task modulate EEG activity (specifically mu rhythm) during actual practice. Moreover, there have been no attempts to determine whether cortical activity changes noted in practice are evidenced in behavioural measures of motor learning, as assessed on a retention test. Therefore, in the present study, we compared short-term physical and observation practice during the acquisition and retention of a novel motor tracing task and evaluated how EEG mu rhythm is moderated during each type of practice. Our primary research question related to whether observational practice brings about change in EEG mu rhythm, comparable to that seen during physical practice, during “practice” of a novel motor task, and during a subsequent postpractice observation phase for both groups. To answer this question, EEG measurements were collected during either physical or observational practice across 45 trials of a flower-tracing task performed using a joystick. We also compared these two practice groups to a third, no-practice control group in a postpractice observation-only session. Relative to resting baseline, we hypothesized that mu rhythm would be suppressed at all the central interpolated channels for both the physical practice and the observation practice groups in comparison to the no-practice group, with greater suppression during physical practice.

Our second research question pertained to whether observational practice brings about behavioural evidence of motor learning, based on comparisons of the three groups in a delayed retention test. This delayed testing under the same conditions is regarded as a critical way of assessing motor learning, such that long-term effects of practice can be ascertained, uninfluenced by temporary factors associated with fatigue, motivation, or the conditions of practice [38, 39]. This delayed retention test was conducted without EEG, but all three groups were compared. We predicted that behavioural performance following physical practice would be improved when measured in a retention test compared to observation practice and no practice. However, if there are benefits to be gained from observation practice, we predicted that this group would perform faster and more accurately than the control group would. We expected that any differences in mu rhythm noted during practice and in the postpractice observation session would be evidenced in behavioural measures of motor learning as assessed on the delayed retention test.

## 2. Materials and Methods

### 2.1. Participants

Thirty healthy individuals between the ages of 19 and 40 years were recruited from the community. Participants were pseudo-randomly assigned to one of three groups with the constraint of *n* = 10/group and equal distributions of males and females (males = 3/group): physical practice (PP) (*M*_age_ = 26.60 yr, SD = 7.18), observation practice (OP) (*M*_age_= 24.4 yr, SD = 3.37), and no practice (NP) control group (*M*_age_ = 27.70 yr, SD = 6.0). All participants were right-handed as confirmed by the Edinburgh Handedness Inventory [44]. They reported normal or corrected-to-normal vision, no motor problems, and no known neurological disorders. The experiment was conducted over two days, and informed consent was obtained from all participants according to the ethical guidelines of the University of British Columbia.

### 2.2. Motor Task

**Figure 1:**
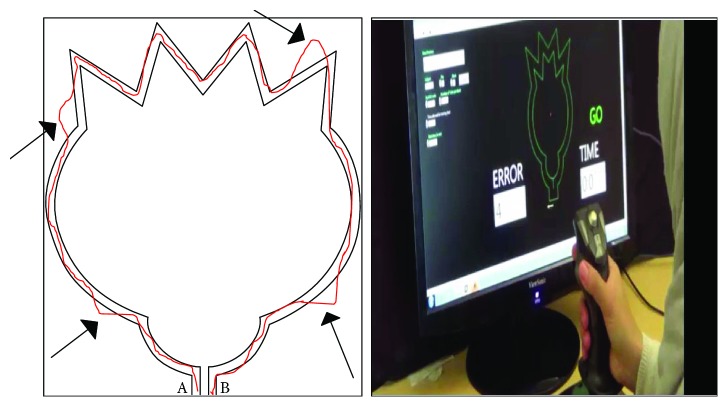
The flower-tracing task. Each arrow represents an error. The total tracing time starts at A and ends at B.

We used a computerized version of the flower-tracing task used in the Movement Assessment Battery for Children (MABC [45]). The flower figure was displayed on a computer screen using custom LabVIEW 7.1 software (National Instruments Co., Austin, TX). Participants were instructed to trace the figure between the two solid lines of the flower figure (Figure 1) as quickly and accurately as possible in a clockwise direction using a joystick. An error was registered each time the participant crossed beyond the two solid lines of the flower. The number of errors and the time it took the participant to complete the trial were displayed on the screen following each trial.

### 2.3. EEG Recording

EEG was recorded using a 64-channel HydroCel Geodesic Sensor Net with a Net Amps 300 amplifier at a sampling rate of 250 Hz via EGI software (Net Station, Electrical Geodesics Inc., Eugene, OR). At the start of the acquisition, impedance values for all EEG channels were less than 50 k*Ω*. The collected signals were referenced to the vertex (CZ).

### 2.4. Procedure

**Figure 2:**
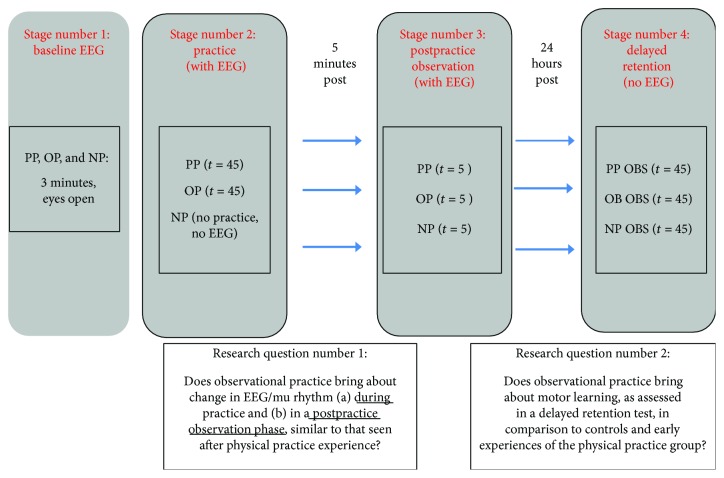
Research design and research questions.

The experiment was divided into four sessions: (1) baseline EEG, (2) practice, (3) postpractice observation, and (4) delayed retention.  Figure 2 shows the design of the experiment as well as the primary and secondary research questions related to the design.

#### 2.4.1. Baseline EEG

Baseline EEG data were first collected on all participants for 3 minutes while they were viewing a blank screen. They were asked to keep their eyes open and to sit still without moving their limbs or eyes.

#### 2.4.2. Practice Sessions

*(1) Physical Practice (PP) Group*. Participants were familiarized with the joystick by tracing a cross on the computer screen for 60 seconds. Once familiar with the movement of the joystick, they performed three blocks of the flower-tracing task for a total of 45 trials. Participants were instructed to trace the flower figure between the two solid lines using the joystick as quickly and accurately as possible. A 2-minute rest was provided between each block of 15 trials. EEG was recorded during all trials.

*(2) Observation Practice (OP) Group*. Participants in this group observed video clips of a model performing the flower-tracing task. The model in the video clips was right-handed and was a novice to the task. She was selected over several performers as she showed the least trial-to-trial variability in performance, yielding the most typical learning curve.

Each video clip represented one trial, with a total of 45 trials. Similar to the PP group, a 2-minute break was provided between each block of 15 trials. The video clips used in the observation trials were recorded at a resolution of 1280 × 720 pixels and a frame rate of 60 Hz. Additionally, the recording was from a first-person perspective, as this results in improved learning [46] and stronger hemispheric activation [47, 48] compared to the third-person perspective. Observers were instructed to refrain from any movement, and their behaviour was monitored via a video camera. EEG was recorded during all trials.

Participants were instructed to pay attention to the model's movement and were told that they would be doing the same task the following day. To ensure that participants were paying attention to the recordings, each participant was asked to state the tracing time or/and the number of errors made by the model at the end of each trial. These questions were randomized so the observers did not identify the pattern and focus on observing one measure on the screen.

*(3) No Practice (NP) Control Group*. Participants in this group did not receive physical or observation training. Participants in this group just completed the baseline EEG trials and the postpractice observation stage (as below).

#### 2.4.3. Postpractice Observation Session

Five minutes after the training session, participants in the PP and OP groups viewed a video of the same model observed by the OP group performing the last five trials of the learning experience. The NP group began the session with the postpractice observation. All participants were instructed to pay attention to the model's performance. EEG was recorded as participants observed the model's movements.

#### 2.4.4. Delayed Retention

Twenty-four hours after the training session, participants in all groups performed 45 trials of the motor task (again with 2-minute rests every 15 trials). No EEG was recorded during the retention session.

### 2.5. EEG Processing and Analysis

EEG data collected from each participant were processed and analyzed using Brain Electrical Source Analysis (BESA) software (MEGIS Software GmbH). Data were first manually screened for eye blinks and eye motion. Trials where eye movement occurred at the time of the task were removed. This resulted in approximately 10% of the total trials from all groups and all conditions being removed from further analysis. Data were filtered at 4–40 Hz and a notch filter of 60 Hz was applied. Independent component analysis (ICA) was then performed on the whole data set using an extended infomax algorithm for mixed sub-Gaussian and super-Gaussian sources in BESA. Using this approach, spatial topographies of the motion artifacts were first defined manually and then the brain signal topographies were determined. The artifact signal at each electrode was reconstructed with a spatial filter taking into account the artifact as well as the brain signal subspace. The reconstructed artifact signal was then subtracted from the original EEG segment.

EEG data were then transformed to a virtual montage using BESA. Virtual montages estimate the voltage at idealized locations of the standard electrodes into 27 channels using spherical spline interpolation. We used the 10-10 virtual standard montage. The average reference was computed for each time point as the mean voltage over the interpolated amplitudes of the standard virtual scalp electrodes. For each clean segment, the power in the 8–13 Hz range was computed using fast Fourier transforms (FFT). The data were segmented into epochs of 2 seconds for each trial in each condition. FFT was performed on the epoched single trials (1024 points) and averaged for each block in each condition to obtain the experimental mu value for each condition. For the baseline mu value, FFT was performed on one minute of the epoched resting state data. Using these values, the Mu suppression index (MSI) was calculated.

### 2.6. Mu Suppression Index (MSI)

We calculated the ratio of the mu power in the experimental condition relative to the mu power in the baseline condition. The ratio is used to control for variability in absolute mu power as a result of individual differences, such as scalp thickness and electrode impedance [49]. Because ratio data are inherently skewed, a log transform was used for the purposes of parametric analysis [49]. The MSI is a change score of absolute mu power (8–13 Hz) between the baseline and experimental conditions. It was calculated as

(1) $$\begin{array}{c} MSI=\log\;\frac{mu\;power\;experimental\;}{mu\;power\;baseline\;}. \end{array}$$

A log value below zero in the area of C3, C4, and CZ indicates mu suppression or activation of premotor or sensorimotor neurons and is considered an index of mirror neuron system functioning. A value of zero indicates no suppression or no change from baseline. Values above zero indicate synchronization or deactivation of the premotor or sensorimotor neurons, perhaps indicating inhibition of these premotor regions.

The MSI was calculated for each participant (using rest as baseline) for the electrodes C3, CZ, and C4. These central electrodes record the activity of the left, middle, and right sensorimotor regions, respectively.

For statistical analysis, we first compared MSI for the PP and OP groups in acquisition (stage 2) across 3 practice blocks (*t* = 15/block) and the three electrode sites. These data were compared in a 2 group × 3 electrode site × 3 block mixed-design ANOVA with repeated measures on the second and third factors. Our primary aim with this first analysis was to assess for group differences as a function of the type of experience (observation versus physical practice) as well as to determine any changes across practice blocks. Second, we compared the MSI values to zero for each electrode site to assess whether there was evidence of suppression for each group. This allowed us to determine at the within-group level, if there was evidence of suppression (and where based on electrode site). As such, we performed single sample *t*-tests (comparing against zero), with Bonferroni-corrected *p* values based on the number of electrodes (.05/3 = .017).

A second analysis was performed on the postpractice observation session (stage 3) for all 3 groups. Again, we first compared across groups (PP, OP, and NP) and electrodes in a 3 group × 3 electrode RM ANOVA, with repeated measures on the second factor. We also compared MSI against zero in single sample *t*-tests for each group and each electrode (again based on Bonferroni-corrected *t*-tests).

### 2.7. Behavioural Measures and Analysis

Two behavioural measures were used to assess learning: (1) error, which was denoted as the number of times the participant crossed out of the flower figure's bounds, and (2) total trace time, which was described as the time it took the participant to complete each trace/trial. These two measures were analyzed in retention testing in a 3 group (PP, OP, NP) × 9 block (5 trials/block), mixed-design ANOVA, with repeated measures on the second factor. Greenhouse-Geisser corrections were applied to violations to sphericity associated with the repeated-measures factor. Significant interaction effects were followed up with Tukey HSD post hoc comparisons (*p* < .05). Due to errors in processing, we were missing data from one control group participant (NP) and from one block of a PP participant; these individuals were not included in the reported statistical analyses. As a check, we re-ran the analysis with the PP participant using estimated values (interpolating based on means for surrounding blocks), and this did not affect the behavioural results.

## 3. Results

### 3.1. Does Observational Practice Bring about Change in EEG Mu Rhythm Similar to That Seen after Physical Practice?

#### 3.1.1. Mu Suppression during Practice for Both OP and PP Groups

**Figure 3:**
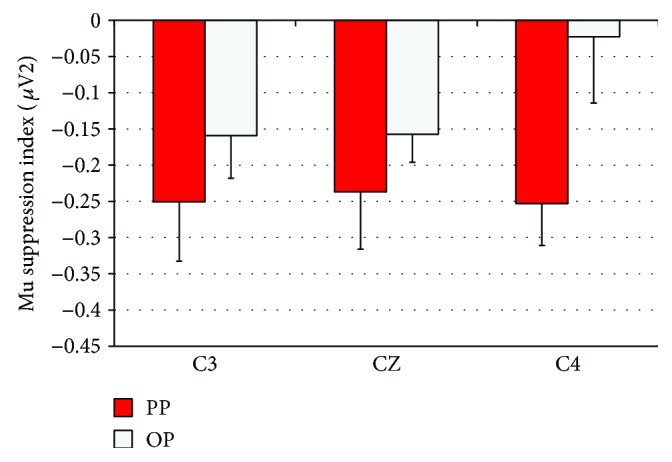
Mu suppression index during physical (PP) and observational practice (OP) (i.e., stage 2 testing) at the central interpolated electrodes C3, CZ, and C4. Values represent the mean log ratio of mu power at the frequency range of 8–13 Hz in the experimental condition compared to baseline. A ratio of negative value indicates suppression. Error bars represent standard error of the mean SE.

In general, mu suppression across the two groups and across practice blocks and electrodes looked similar. The results of the mixed ANOVA on MSI showed neither significant main effects nor significant interactions (all *F*s < 1.37, except the group main effect, where *F*(1, 18) = 2.78, *p* = .11, *η*_*p*_^2^ = 0.13). In Figure 3, we have plotted the MSI for the PP and OP groups for each of the three electrodes averaged across all trials. Based on comparisons to zero, there was evidence of bilateral mu suppression during PP, but only left lateralized mu suppression during OP. This was confirmed through single sample *t*-tests for both the PP and OP groups for each electrode, as displayed in Table 1. During PP, the average MSI at C3, CZ, and C4 was significantly less than zero. During OP, the average MSI at C3 and CZ was significantly less than zero, but it was not at C4.

#### 3.1.2. Mu Suppression during the Postpractice Observation Phase

**Figure 4:**
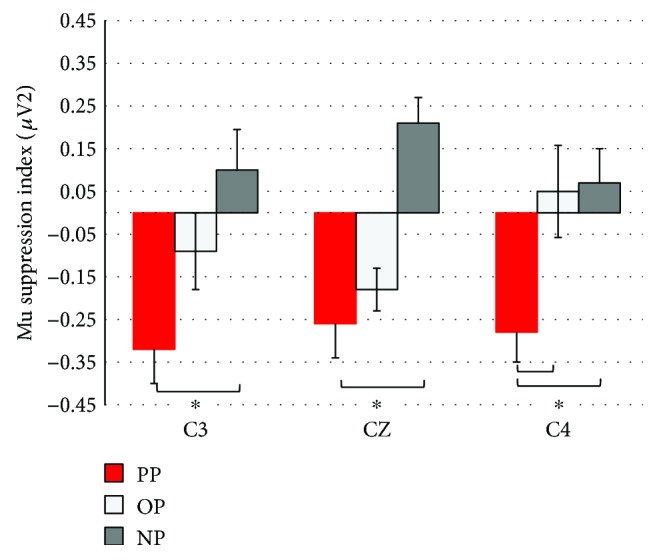
Mu suppression index for the PP, OP, and NP groups during postpractice observation (stage 3 testing). Error bars represent standard error of the mean (SE). Asterisks indicate significant between group differences (*p* < .05).

Comparison of the three groups during postpractice observation yielded a significant group main effect, *F*(2, 27) = 9.68, *p* = .001, *η*_*p*_^2^ = 0.42, but no electrode effect (*F* < 1). In Figure 4, we have plotted the MSI values for each electrode as a function of group. Based on Tukey HSD post hoc comparisons, the PP group showed more overall suppression than both the OP and NP groups and the OP group showed more suppression than the NP control group did. The interaction between group and electrode approached conventional levels of significance, *F*(4, 54) = 2.52, *p* = .051, *η*_*p*_^2^ = 0.26. As can be seen in Figure 4 and based on Tukey post hoc comparisons, the PP group displayed significantly greater MSI compared to the NP group at all electrodes (i.e., strong bilateral activation across both hemispheres). However, MSI values of the PP group were only significantly higher than were those of the OP group at C4, in the right hemisphere. An additional analysis was run on the baseline mu results for each electrode to test for pre-existing group differences, in a 3 group × 3 electrode RM ANOVA. There were no group differences in baseline mu all group *F*s < 1. Group mean values varied from 6.7 to 12.2.

To test for evidence of suppression, single sample *t*-tests comparing the MSI value to zero for each group and electrode showed that for the PP group, MSI at all three electrodes (C3, CZ, and C4) was significantly less than zero (*p* < .006). For the OP group, only the middle, central electrode, CZ, had a significantly lower MSI value (*p* = .004). For the NP control group, MSI was significantly higher than the baseline at CZ (*p* = .003).

### 3.2. Does Observational Practice Bring about Motor Learning?

**Figure 5:**
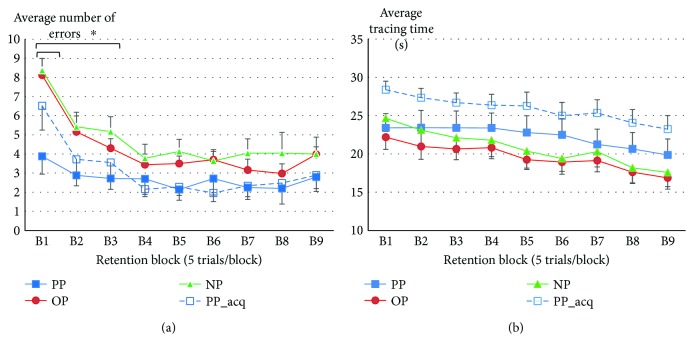
(a) Average number of errors across 9 blocks of retention testing (*t* = 9/blk) for the physical practice (PP), observational practice (OP), and no practice (NP) groups and first-day acquisition trials for the PP group (PP_acq). Error bars represent standard error (SE). ^∗^PP group significantly different to NP group B1–B3. PP group significantly different to OP group (B1). (b) Average tracing time across 9 blocks of retention testing (*t* = 9/blk) for the physical practice (PP), observational practice (OP), and no practice (NP) groups and first-day acquisition trials for the PP group (PP_acq). Error bars represent standard error (SE). There was no interaction.

In Figure 5(a), we have plotted the average number of errors for the three groups during the nine blocks of testing for the delayed retention test. For illustrative comparison, we have also plotted the acquisition data for the PP group. There does not appear to be any savings associated with previous observational practice for the OP group, when comparing their performance to the PP and NP groups. The PP group had the fewest number of errors in retention, particularly across the first few retention blocks, but across groups, number of errors decreased across blocks. With respect to statistical confirmation of the descriptive data, although the main effect of group was not significant, *F*(2, 25) = 2.88, *p* = .075, *ɳ*_*p*_^2^ = 0.19, there was a significant block, *F*(5.05, 126.23) = 24.04, *p* < .001*ɳ*_*p*_^2^ = 0.49, and a significant group × block interaction, *F*(10.10, 126.23) = 2.21, *p* = .021, *ɳ*_*p*_^2^ = 0.15. The block effect comprised significant linear and quadratic trend components (*p* < .001). Tukey's post hoc analysis of the interaction showed significant differences between the PP and NP group for blocks 1, 2, and 3, with the PP group showing fewer errors than the NP group. The PP and OP groups were only significantly different on block 1. However, there was no significant difference between the OP and NP groups for any practice block.

**Figure 6:**
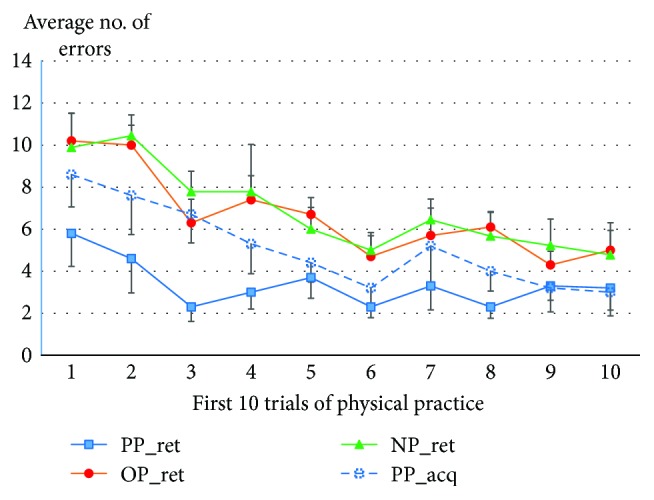
Average number of errors across the first 10 trials of physical practice in retention for the physical practice (PP), observational practice (OP), and no practice (NP) groups as well as during first-day acquisition for the PP group (PP_acq). Error bars represent standard error (SE). There were no group differences comparing PP_acq to the OP_ret and NP_ret.

We have also plotted data for the first 10 trials of physical practice for all groups in order to better describe group effects and illustrate how differences in performance were not apparent on these first 10 trials, but rather emerged with practice. These data are shown in Figure 6 (i.e., first 10 trials of retention for all groups in addition to the first 10 trials of acquisition for the PP group). A group × trial ANOVA comparing the PP group during acquisition and the NP and OP groups during retention confirmed the absence of any group effect, *F*(2, 26) = 1.30, *p* = .29, *ɳ*_*p*_^2^ = 0.09, nor a group × trial interaction (*F* < 1).

For tracing time, the data are illustrated in Figure 5(b), again with the PP group's acquisition data illustrated for comparison purposes. Although there was still a main effect of block, *F*(3.08, 77.01) = 12.25, *p* < .001, *ɳ*_*p*_^2^ = 0.33, which comprised a significant linear trend component (*p* < .001) due to decreasing times as retention testing continued, there was no group or group × block interaction (both *F*s < 1).

## 4. Discussion

This study is the first to investigate how mu suppression changes as a function of both observation and physical practice and what this means in terms of behavioural indices of motor learning. As predicted, the behavioural results showed that during the retention phase, the PP group made the least number of errors compared to the OP and NP control groups. However, the groups did not differ in average tracing time during retention. This suggests that benefits from physical practice were mostly a result of spatial accuracy improvements, rather than speed. Although both the PP and OP groups showed evidence of mu suppression, in both practice and postpractice observation (especially when compared to the control group), the patterns and degree of suppression differed across the groups. Therefore, although there was evidence of suppression in the OP group, suggestive of changes in cortical processing due to observational practice experience, these patterns of activation did not appear to manifest in improved motor learning, when the OP group was compared to the NP control and PP groups on a delayed retention test.

With respect to the behavioural data, there was no evidence of motor learning in the OP group. For tracing times, there were no significant group-related differences. There was no evidence that prior physical practice resulted in tracing time benefits. Rather, there was a trend for the PP group to be slower than the NP and OP groups. However, there was also no evidence that prior observation aided performance times, when comparing the NP and OP groups. The behavioural effects related to prior physical experience were evidenced in tracing errors. The PP group showed fewer errors in retention during the first five trials, when compared to both the NP and OP groups and across the first 15 trials when compared to the NP group. However, the OP group did not differ statistically from the NP group. Because accuracy could be compromised as a function of speed, it may be that the slower tracing times for the PP group (at least descriptively) were compensated for by fewer errors. We do not have a good reason why the PP group might have weighted accuracy more highly than tracing time. We would have expected the NP group in retention to have looked like the PP group in acquisition, but as can be seen from Figures 5(a) and 5(b), the PP group showed fewer errors and longer tracing times right from the start.

The lack of differences between the NP group and the PP and OP groups for the behavioural measures of motor learning might be due to the five 30–40 s observation trials shown during the postpractice observation testing session. It is possible that learners did not need to observe all 45 visual trials to show gains in subsequent execution and that these five trials of “good” performance may have been adequate to bring about performance benefits in retention, at least with respect to tracing time. If so, this suggests that covert practice benefits were likely more strategically mediated, associated with familiarity with the flower shape, rather than a motor-mediated strategy based on action simulation. This does not mean that there was no action simulation, as appeared to be evidenced by the EEG mu suppression data, but that this suppression or simulation was not associated with behavioural indices of motor learning.

The neurophysiological results showed that, compared to baseline, mu rhythm was significantly suppressed over both hemispheres during PP and only over the left hemisphere during OP. However, the magnitude of this suppression did not change, as a function of practice. This result is not consistent with studies that reported changes in the magnitude of MNS activity during practice. Nakano et al. [37], for example, reported a significant difference in suppression between the first and last of 5 trials of observation of a ball rotation task. Although the authors associated this decrease in suppression with motor learning, it is difficult to draw a conclusion about motor learning based on so few trials (in our study, there were 45 trials of practice). The unchanged magnitude of mu suppression is also not in line with the neural efficiency hypothesis, which associates improved learning with less cortical activation [50]. Several lines of evidence show that experts exhibit less suppression during the execution and observation of motor skills, suggesting that more experience eventually leads to a more efficient neural processing [43]. However, contrary to our study, which focused on short-term practice, these studies tested individuals with years of experience and researchers have identified different brain networks, with different activation patterns, involved in short-term and long-term motor practice [51].

As expected, mu suppression during physical practice was bilateral, showing that both hemispheres were active during movement performance. Contrary to our hypothesis, however, mu suppression during observational practice was higher in magnitude in the left hemisphere compared to the right hemisphere and hence did not show a similar bilateral suppression as noted for physical practice. Given that both the model and the study participants were right-handed and that the observers watched the movement from a first-person perspective, bilateral suppression of mu rhythm would be expected based on previous work [52].

Despite this research above, which was primarily based on observation of already experienced actions, there is evidence that action observation can lead to a more lateralized activation pattern. For example, Perry and Bentin [53] showed that when they examined right-handed participants while observing movements of both right and left hands from a first-person perspective, mu suppression was evidenced at the frequency range of 8–13 Hz. This suppression was stronger in the hemisphere contralateral to the hand being observed compared to the hemisphere ipsilateral to the observed hand (similar to our data). Similarly, when Quandt et al. [54, 55] presented video clips of a right-handed model from a first-person perspective, action observation was associated with greater suppression at the alpha frequency range in the left hemisphere compared with the right hemisphere. In a different study, when a third-person, action-observation paradigm was employed, suppression was greater over the right hemisphere compared with the left hemisphere [55]. The left-lateralized activation reported in this study is also consistent with fMRI studies. These studies have revealed that watching right-hand reaching-and-grasping movements from a first-person, egocentric perspective elicited larger BOLD responses in the left anterior intraparietal cortex of right-handed observers [17]. This contralateral effect, however, was replaced by an ipsilateral response (i.e., the right hemisphere) in the anterior superior parietal lobule when the right-handed observers viewed the right-handed movements from an allocentric perspective (i.e., facing the model) [56].

Complementing the EEG differences in practice between OP and PP, the postpractice observation testing session also yielded differences between groups based on experience. Although for the OP group, these postobservation trials had already been viewed, whereas these were novel videos for the PP and NP groups, there was no suggestion that the patterns of activation were affected by novelty. There was no significant block effect in acquisition, such that the patterns of activation shown in the first block of practice were maintained throughout practice. Moreover, patterns of activation seen for the PP group while acting were also maintained for this group while observing. Again, this suggests that prior experience, rather than novelty or familiarity, was responsible for these effects.

For the PP group, postpractice observation was characterized by bilateral suppression. For the OP group, observing the same stimuli as the PP group, action observation was characterized by left-lateralized suppression. It appears that observation without any physical practice activates only a subset of brain regions, which also explains the lateralized effect during observation practice. The absence of suppression for the NP group, however, suggests that more than 5 trials of observation are needed for this suppression to be evidenced or that a learning model who shows improvement across trials is necessary for suppression. The model shown in the postpractice observation session only was of near perfect performance (i.e., the last five trials from practice). Behavioural studies that have compared expert and learning models have provided evidence that watching learning models engages the observer in a problem-solving mode in which he/she considers all the relations between the movement patterns and their outcomes to optimize performance [57]. Given the association between the left hemisphere and relational reasoning [58], the left-lateralized suppression during visual training could be moderated by model type, which continues to influence patterns of activation during near-perfect performance. In light of this explanation, it is possible that the bilateral effect during observation reported in other studies in this area was because the observers viewed error-free hand movements, with no learning component.

What is important from the postpractice observation data is the fact that differences existed between the groups based on the types of experience, despite the fact that all three groups were watching the same action stimuli and that we only collected data from 5 trials in practice (each trial lasting ~60 s). Differences in activation between the OP and PP groups might simply be a carryover effect from practice, such that whatever areas of the brain were activated during initial exposure continued to be activated during a subsequent observation phase. Because the observe-only group never physically practiced and did not show bilateral suppression, there was no reason to think that this would be observed postpractice as nothing had changed. For the PP group, it appears that it was able to resonate and engage in action simulation, based on previous physical experience with the task, such that the patterns of activations resembled these early learning physical practice experiences. In comparing the size of suppression and making conclusions about motor-mediated learning in observation conditions, the PP group exhibited the strongest suppression compared to the OP and NP control groups. This suggests that for observation to induce significant suppression, it has to be preceded by active motor experience with the motor skill of interest. This result corroborates with EEG studies that have stressed the role of prior active motor experience (long- or short-term) in modulating mu responses during observation [59].

Although previous PP in this study involved both hemispheres and led to the strongest suppression, comparable suppression between OP and PP groups was shown at the medial central site (i.e., CZ), especially in comparison to the NP control group. Interestingly, although the NP control was not that different to the PP and OP group with respect to their behavioural performance in retention, at least with respect to tracing times, the five trials of EEG collected during the observation-testing session revealed significant differences between these groups. No suppression was observed at all three central sites (in fact, there was significant synchronization in comparison to baseline). Even though the participants in the control group were observing a motor task, their preceding lack of experience with this task either covertly or overtly moderated any motor system suppression at this central location. Therefore, action-observation does not always induce suppression, supporting the suggestion that the MNS and mu suppression more specifically is sensitive to previous experiences with the task, both visual and physical (see also [60]). Prior physical and observation practice experiences caused mu suppression in our study, but to different degrees and in qualitatively different ways.

This study has some limitations that deserve mention. One of these concerns potential accuracy-speed trade-offs. Any improvement in one measure could be attributed to a decrease in the other. Although not reported, correlations between these two measures in retention were all small (*r*s < 0.25) and nonsignificant. However, in future work, fixing one measure of the task to examine the changes in the other would rule out any possible trade-off influences and narrow down alternative explanations for the observed effects (e.g., requiring zero-error performance).

Monitoring the observers' motion only via a video camera, without a stringent control for muscle activation, introduced another limitation. Although the activation during PP was different from that during OP, there is still a possibility that any mu suppression could be due to muscle activation. To avoid such confounding effects, electromyography (EMG) should be used to accurately detect any possible movement.

The lack of change in the magnitude of mu suppression throughout both types of practice could be a result of the number of training trials (i.e., 45 trials). This number was used because in pilot testing, it was shown that behavioural measures of performance leveled out (i.e., plateaued) around the 45th trial. Increasing the number of trials could also cause participants possible discomfort with the EEG net, which in turn could negatively impact performance. Nevertheless, in future work it would be important to increase the amount of practice, especially as behavioural plateaus do not indicate the absence of learning [61]. Moreover, it may be that for observational learning benefits to be realized in this task, observational practice trials should be increased. Although behaviourally, the OP group did not look different to the NP group, which might suggest that five observation trials were enough to bring about some performance gains, watching 45 trials of practice from a learning model did lead to differences in cortical activation. In future work, it will be important to test motor performance of a no-practice control group in the absence of any observational practice trials to better appreciate the short-term effects of watching and the volume of practice which is needed to bring about observational-related changes.

Finally, although mu rhythm is mainly described as EEG oscillations at the frequency range of 8–13 Hz, some authors limited mu rhythm to the frequency band of 8–10 Hz (e.g., [26]). EEG researchers have identified two frequency ranges within the alpha range (8–13 Hz): the lower alpha (8–10) Hz and the upper alpha (10–13) Hz. The lower alpha emanates from the somatosensory cortex and is modulated by motor activity, showing a more anterior and asymmetrical hemispheric effect. The upper band, in contrast, consists of posterior bilateral waves, which cluster mainly around the parietooccipital cortices and is primarily modulated by visual stimulation [62]. Given that the observed suppression in this study could be a result of visual stimulation or motor activation or both, examining each component separately would shed more light on both the hemispheric activation and the source of stimulation. It may also be of interest in future work to conduct time frequency analysis on the EEG, to help provide more information about the complex network dynamics underlying observational practice [63].

In conclusion, we have demonstrated that observation practice induces neurophysiological changes as indexed by mu suppression at central sites, which provides evidence for motor-based processes during observational practice. However, there was no evidence that these motor-related processes were related to motor learning and behavioural measures of learning in retention. The lateralized suppression during observation practice suggests that cortical processes involved in this covert type of practice might not be entirely motor-based and that the lateralized activation during OP and the bilateral activation during PP at the central sites suggest that OP does not trigger all brain areas activated during PP. Therefore, observation practice cannot replace physical practice, even though in some instances there may be benefits to be gained behaviourally from this type of practice (at least in comparison to not practicing). Because of EEG differences between OP and NP control conditions during a postpractice observation phase, there is evidence that OP is leading to neurophysiological changes, although we did not have evidence that this suppression was linked to motor learning outcomes.

Importantly, we confirm the vital role of previous motor experience in modulating mu responses during observation, suggesting that employing movements that are within the observer's motor repertoire (i.e., prior physical exposure) is more likely to result in optimal activation during a subsequent practice phase. To a lesser extent, observers without this experience could benefit from watching movements where they have only had previous visual experience. Although both physical and observational practice might share some similarities, the underlying mechanisms by which each of them operates appears to be different both qualitatively and quantitatively. To better understand the relationship between mu responses and motor learning during observational practice, researchers should address other factors that could influence this relationship, such as handedness, observation perspective, the amount of visual familiarity, model's expertise, and the type of motor task. Further study of the relation between these types of practice and their neurophysiology would help to elucidate on the dominant mechanisms underpinning observation practice and the conditions which maximize motor-mediated learning under these conditions. It has yet to be shown that an increase in motor-related areas during observation practice is responsible for better learning. One possible future method to help determine how cortical activation in motor-related areas of the brain relates to learning is to use methods to stimulate the brain either during or before a period of observational practice, potentially through transcranial direct current stimulation (tDCS).

## Figures and Tables

**Table 1:** Single sample *t*-test results (and Cohen's d), comparing MSI to zero for each electrode site for PP and OP groups during the stage 2 practice session.

	PP				OP			
	df	*t*_obs_	d	*p*	df	*t*_obs_	d	*p*
Electrode								
C3	9	−3.06	0.97	.007^∗^	9	−2.71	0.86	.012^∗^
CZ	9	−3.01	0.95	.008^∗^	9	−4.08	1.2	.002^∗^
C4	9	−4.39	1.3	.001^∗^	9	−0.25	0.08	.405

^∗^Statistical significance based on Bonferroni-corrected alpha values (.05/3 = .017).
